# Physico-Chemical Characteristics and Culturable Microbial Communities of Grape Berries Change Strongly during Noble Rot Development

**DOI:** 10.3390/plants9121809

**Published:** 2020-12-21

**Authors:** Júlia Hegyi-Kaló, Ádám István Hegyi, József Geml, Zsolt Zsófi, Xénia Pálfi, Kálmán Zoltán Váczy

**Affiliations:** 1 Food and Wine Research Institute, Eszterházy Károly University, 6 Leányka Street, H-3300 Eger, Hungary; hegyi-kalo.julia@uni-eszterhazy.hu (J.H.-K.); hegyi.adam@uni-eszterhazy.hu (Á.I.H.); zsofi.zsolt@uni-eszterhazy.hu (Z.Z.); palfi.xenia@uni-eszterhazy.hu (X.P.); 2 Department of Microbiology and Biotechnology, SZIU, 14-16 Somlói Street, H-1118 Budapest, Hungary; 3 MTA-EKE Lendület Environmental Microbiome Research Group, Eszterházy Károly University, 6 Leányka Street, H-3300 Eger, Hungary; jozsef.geml@gmail.com

**Keywords:** *aszú* berries, botrytization, compression test, texture analyses, Tokaj

## Abstract

*Botrytis cinerea* is a well-known pathogen of grapevine. However, under certain microclimatic conditions, *Botrytis* infection results in noble rot, an essential process in the production of the world-known Tokaji *aszú* wines in Hungary. We investigated the physico-chemical characteristics and culturable microorganisms associated with grape berries through several noble rot phases in the two main cultivars grown in Tokaj: *Vitis* *vinifera* cv. “*Furmint*” and “*Hárslevelű*”. We measured physical and analytical parameters routinely tested in viticulture and analyzed the ITS rDNA sequence data of fungi isolated from the sampled berries. We observed significant differences in the physico-chemical parameters among the noble rot phases as well as sampling dates. The greatest variation in berry texture and microbial structure was observed in the initial phases, with variables converging as the noble rot progressed. By finding a bijection between the examined chemical properties and the factorial parameters (e.g., noble rot phase, collection time, cultivar), an appropriate sweet winemaking material can be designed. Fungal community differed significantly among cultivars, with higher number of species observed in *Hárslevelű*. Our results reveal that there is more to noble rot than only *Botrytis* *cinerea* and other microorganisms may play important roles in the *aszú* process.

## 1. Introduction

Noble rot development in grape berries has been used for sweet wine production for centuries. For example, the world-renown Tokaji *aszú* wine is made from noble rotten grapes in Tokaj, Hungary, with *Furmint* and *Hárslevelű* being the most susceptible grape varieties for the botrytization [1,2]. The Hungarian *aszú* winemaking method is different from other botrytized sweet wine making techniques around the world because Hungarian *aszú* wine is made from grapes that are selected and picked by hand throughout the long noble rot process [3,4]. In grapes, the pathogen *Botrytis cinerea* mostly causes grey rot of berries, but under certain microclimatic conditions, infected berries go through the process of noble rot [5,6]. *B. cinerea* is a common and widespread ascomycete fungus and necrotrophic pathogen attacking numerous different plant species (e.g., grape, tomato, kiwi fruit, strawberry and raspberry, herbaceous, shrub and tree species) [7,8,9]. Fournier et al. [6] compared populations of *B. cinerea* associated with grey mold versus noble rot symptoms in various wine regions of France and showed that noble rot development was influenced by climate, cultivar, vintage, cultural practices, and berry microbiome [10]. The occurrence of noble rot is dependent mainly on microclimatic conditions [11]; however, there is a lack of information on the textural and microbial characteristics of the grape berries during the noble rot process.

Carbajal-Ida et al. [12] analyzed the physico-chemical properties of botrytized *Chenin Blanc* grapes and found that textural characteristics changed during the botrytization process and are influenced by cultivar, vintage, and the time of harvest [13]. Similarly, changes in grape berry texture parameters, particularly in berry skin and peduncle, have also been reported during the development of grapes for ice wine making [14]. It is well-known that the process of botrytization results in a unique sweet wine product [4,15], which is very different from ice wine. Ice wine is made from grapes that are naturally frozen on the vines and have a high sugar content [16], but in many cases, the organoleptic sense of sweetness is disharmonic because of the lower sensory acidity. Noble rot wines also contain high sugar content [17], but the titratable acidity is higher and the resulting sense is more balanced [18]. The reason for this balance is that the water naturally evaporates gradually from the grape berries during the noble rot, but in the case of ice wine making, the principal cause of water loss is freezing, not gradual evaporation [3,19].

The microbial community of the over ripened and ice wine grape berries is very diverse, but usually *B. cinerea* does not play a role in the microbial composition of the ice wine [20]. Conversely, *B. cinerea* characteristically occurs in a high percentage on the surface of noble rotten grapes and is accompanied by many other microorganisms that can contribute to the noble rot process [21,22]. Previous research on the microbial community of healthy grape berries in different grape ripening stages show that fungi mainly appear on the grape berries surface after veraison (i.e., the onset of ripening), and that their importance is limited to the beginning of the fermentation, as later in the fermentation process, the alcohol concentration becomes lethal to most fungi [3,17,23,24,25]. The most important yeast of the fermentation is the *Saccharomyces cerevisiae*, but it rarely appears naturally on the grape surface, only later during the winemaking process [24,26]. The healthy grape surface fungal community is rich in filamentous fungi [27], but the commonly held view is that during the noble rot process, filamentous fungi in *aszú* berries start to decline as *B. cinerea* becomes dominant [28]. Conversely, plant pathogenic fungi are more diverse and abundant on diseased grape berries than on healthy or noble rotten berries [29]. In addition, the effects of vine growing region (terroir), grape cultivar, and the vintage on the microbial community of the grapes have been shown [30]. Despite these earlier works, several important questions remain unanswered related to the relationships between the physical, chemical, and microbial characteristics of grape berries and their interaction during the maturation and noble rot process.

## 2. Results

In 2017, several botrytized berries were collected to characterize the relationships among the physical-chemical properties and microbial communities of grapes during the noble rot process. The berries were sampled at three different times during the harvest period and were classified into four rotting phases based on characteristic morphological features as described in Hegyi-Kaló et al. [28]. We sampled grapevine cultivars commonly used for making aszú wines: *Vitis vinifera* cv. *Furmint* and *Hárslevelű*. Statistical analyses were conducted as follows to characterize the relationships among the different parameter groups and to test for significant variation in each.

### 2.1. Changes in Physical, Chemical and Microbiological Properties during Noble Rot

**Figure 1:**
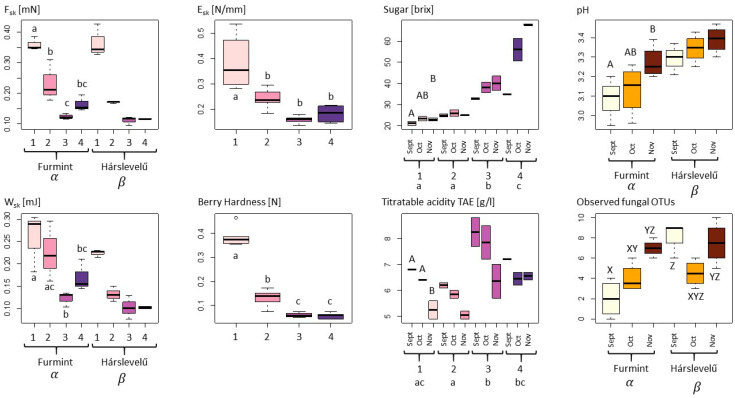
Physical, chemical, and microbiological variables of grape berries undergoing noble rot compared with ANOVA according to noble rot phase (Arabic numerals from 1 to 4), sampling dates (September–November), and cultivars (*Furmint* and *Hárslevelű*). For each dependent variable, only the best fit model showing the most influential categorical variables is shown as selected using AIC criteria (for models, see Table 1). Letters indicate significant differences in post-hoc Tukey HSD tests (*p* < 0.05), with lowercase letters referring to phases, uppercase letters to collecting months, Greek-letters to grapevine cultivars, and XYZ letters referring to the categorical variables of the sampling dates–cultivars interaction model. The color pallet pink to purple indicates the four phases of botrytization, in the subfigure pH and “Observed fungal OTUs” white, orange, and brown means the collection times in order: September, October, and November.

With respect to the measured physical variables, we observed a strongly significant decline in berry skin brake force (Fsk, in mN) and berry skin break energy (W_sk_, in mJ) during the noble rot process in both cultivars, with significant differences also observed between the cultivars (Table 1, Figure 1) [31]. This latter may be driven by the fact that decreases in both F_sk_ and W_sk_ were more gradual in *Furmint*, with a perceptible, but non-significant increase between phase 3 and phase 4, while the measured properties showed a steep decline already by phase 2 and remained low in *Hárslevelű*. Despite these moderate differences among cultivars, the pattern of F_sk_ during the process of the botrytization is very similar in both cultivar (Figure 1).

The elastic modulus of berry skin or skin elasticity (E_sk_, in N/mm) and berry hardness (BH, in N) showed similar patterns to those observed above, with the exception that differences between the cultivars were negligible, with only the noble rot phases having a significant effect on them. Overall, the drop−like decrease between phase 1 and phase 2 can be regarded as an indicator of the beginning of the rotting process (Figure 1).

Regarding the chemical properties, sugar content (soluble solids in brix) was strongly influenced by the interaction of noble rot phase and sampling month (Table 1). The emerging trend is an overall increase in sugar content during the noble rot process, upon which superimposed is another increasing trend during the harvest period from September through November, particularly in phase 3 and phase 4. Values in the first collection time are different from those in the third sampling, with the second sampling being an intermediate. In the case of the phases, three different groups were identified: phase 1 and 2, phase 3, and phase 4. In phase 1, there were no significant differences in the first and second collection time, but there was by the third sampling, which gives a higher basic sugar level for botrytization. If we look at the variation of the sugar levels in phase 4 *aszú* berries, we can see that in October, a quite high sugar content can be reached, although with greater spread in values (Figure 1). The sugar content in collected phase 4 *aszú* berries was highest in November and with small variation, which gives the highest quality berries for *aszú* wine in general.

There were significant differences in pH among the cultivars, with *Furmint* generally being somewhat more acidic than *Hárslevelű*. In addition, there was a significant effect of sampling month in *Furmint*, which showed a continuous increase in pH during the harvest period. No significant differences were found among the noble rot phases (Figure 1).

Titratable acidity tartaric acid equivalent (TA, in g/L (TAE)) differed significantly among noble rot phases, where phase 2 was significantly lower than phases 1, 3, and 4. TA decreased with sampling month in all phases, with the strongest decrease observed in phase 1, where TA values for September and October were significantly different from November. However, although TA levels are high in grape berries that are botrytized early, already in September, acidity is decreased in *aszú* berries in October and November, similar to the starting levels in phase 1 (Table 1 and Figure 1). It is essential to say that higher initial TA indicates a higher quality of noble rotten berries that give the sensory balance to sweet wines.

**Figure 2:**
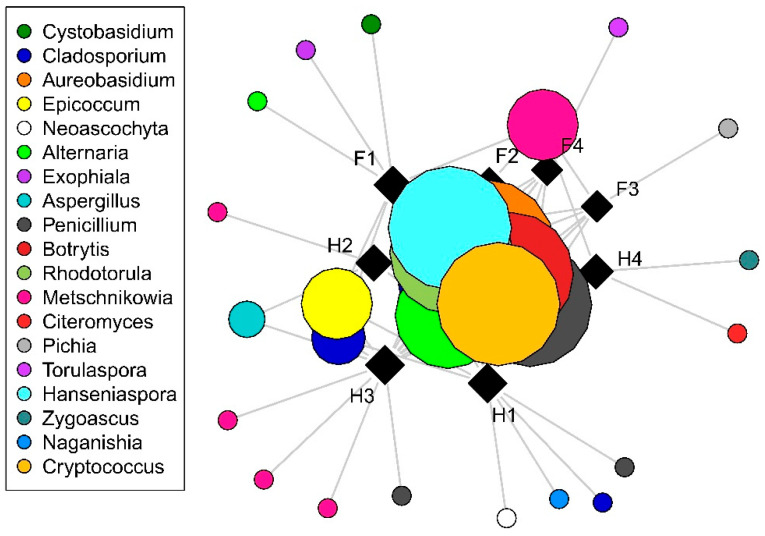
Distribution of fungal operational taxonomic units (OTUs) among cultivars and noble rots phases as visualized by network analysis. Squares and circles indicate samples and OTUs, respectively, with size of circles is proportional to incidence (the number of samples in which the OTU is presented). Abbreviations: F–*Furmint*, H–*Hárslevelű*, with numbers indicating noble rot phases.

With regard to culturable fungi, DNA sequences were generated from a total of 198 isolates and were grouped into 44 operational taxonomic units (OTUs). Of these, 28 OTUs (15 filamentous fungal and 17 yeast OTUs), representing a total of 160 sequences, had ˃80% sequence similarity to a fungal reference sequence and were used for subsequent analyses. Only filamentous fungal richness differed significantly among cultivars, with higher richness observed in *Hárslevelű* (Figure 1). Significant differences in richness were detected in yeasts and filamentous fungi in the interaction case of collection times and cultivars (Table 1). This was driven by the significant increase in fungal richness in *Furmint* from September to November, but not in *Hárslevelű*. No differences in richness were found among the noble rot phases. The network graph reveals the core grape berry microbiome and illustrates the dominance of the genera *Aureobasidium*, *Botrytis*, *Cladosporium*, *Hanseniaspora*, *Metschnikowia*, *Penicillium*, and *Rhodotorula* (Figure 2), while the full list of genera is shown in Table 2.

### 2.2. Relationships among Physical, Chemical, and Microbiological Variables and Noble Rot Phases

**Figure 3:**
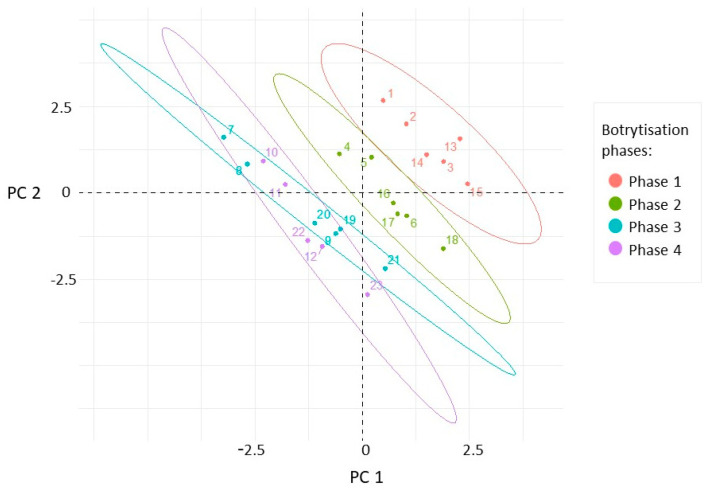
Scatterplot of the samples in the plane defined by the first two principal components calculated from the four textural (F_sk_, E_sk_, W_sk_, BH), three analytical (Sugar, TA, pH), and fungal richness variables. The sample points are colored in accordance with the botrytization phases.

In order to explain the differences between our berry samples, which were selected according to the factors of noble rot phases, collection times, and cultivars, principal component analyses were performed to provide partial visualization of the dataset in reduced dimensions. Three principal components with eigenvalues higher than 1 were obtained. From the variables, PC1 (26.27% total variance) was most highly correlated with TA, BH, and Es_k_. Berry skin break force, sugar content, and W_sk_ dominated in PC2 (22.2% total variance). The projection of the samples along the directions was identified by the first two PCs (Figure 3). The separation of different categories of samples from this scatterplot indicates that the first three botrytization phases were separated and there was an overlap between the third and fourth phases. In the case of sample collection time and grape cultivars, our samples oriented in overlapped frames in this plane (Figures S1 and S2 in the Supplementary Materials).

**Figure 4:**
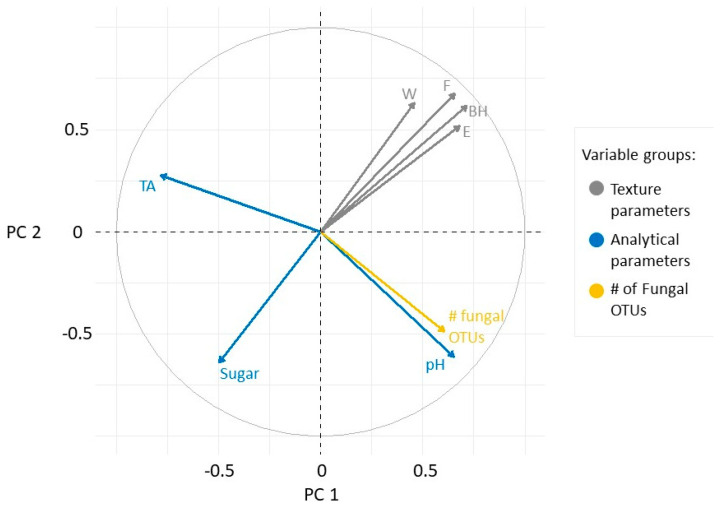
Loading plot of the measured variables in the PC1-PC2 plane. The variables are grouped as texture parameters (grey), analytical properties (blue), and microbial richness (yellow).

Berry texture indicators appeared to correlate positively as they were located at the top of the first quadrant (Figure 4). The pH and the number of fungal OTUs did not correlate with the texture parameters, but did correlate with each other. Sugar was negatively correlated with berry texture indicators, while acidity had no correlation with any other variables.

In order to obtain a comprehensive picture on the distribution of the samples in the observed multidimensional space, partial least square regressions were made and are illustrated in the first two variable planes (see Figure S3 in Supplementary Materials). From this point of view, the situation is the same regarding on botrytization phases, where phases 1, 2, and 3 are separated, but phase 4 overlaps with the third.

**Figure 5:**
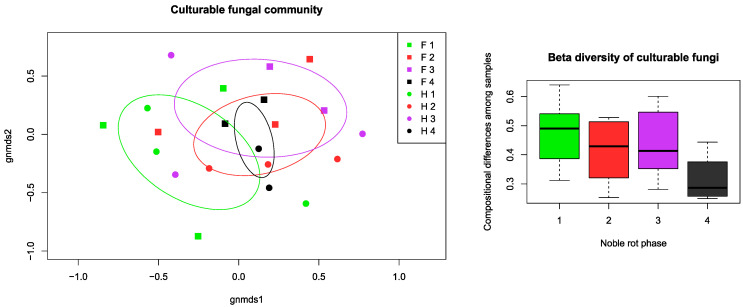
Community composition and turnover of the culturable fungal community isolated from grape berries representing the four noble rot phases discussed in the text. Non-metric multidimensional scaling (NMDS) ordination plot illustrates the compositional differences among all samples, while the beta diversity box plots show compositional differences among samples within noble rot phases. For each phase, ellipses indicate the standard deviation of point scores. Abbreviations: F–*Furmint*, H–*Hárslevelű*.

Aside from the grouping and factor analysis of the samples, permutational multivariate ANOVA (PerMANOVA) was made to compare the dispersion of point scores and centroids of the sample groups concerning factorial parameters. According to the PerMANOVA results, the variable groups of texture parameters, analytical properties, and fungal community were significantly different in distribution between botrytization phases, so the defined phases could be separated by all the measured data. The sample collecting times had a significant effect on the physical properties and fungal richness, but not on the chemical properties. There was an observable community structure of culturable fungi among the noble rot phases as well as among the cultivars. Noble rot phase explained 21.49% (*p* = 0.031), collection time explained 22.23% (*p* = 0.014), and cultivar 24.82% (*p* = 0.014) of the variation, as indicated by PerMANOVA. This pattern was even more pronounced when OTUs were merged at the genus level, with noble rot phase explaining 23.57% (*p* = 0.015) and cultivar 23.62% (*p* = 0.008) of the variation. In addition, we observed lower compositional turnover (beta diversity) among samples of fully botrytized berries in phase 4 than in the partially botrytized berries of the preceding phases (Figure 5).

In Table 3, the significance *p*-values and explained variance showed that all the physical, chemical, and microbial variable groups had significant differences among the four botrytization phases. For texture and analytical parameters, relatively high explained variance values were given (57% and 72%, respectively), highlighting their importance in the process of noble rot. In the case of sample collection time, texture and fungal community had higher and analytics had lower correspondence, but it is necessary to declare, as is shown in Figure 1, that collection time plays an important role in the chemical properties of fully botrytized berries. Finally, cultivars do not have strong influence on the texture and measured chemical properties, but cultivar has a significant effect on fungal community composition, similar to what has been observed by Bokulich et al. [30,32] in non-botrytized grapes.

We used Mantel tests to detect whether possible correlation could be detected between variable groups. In the case of physical and chemical parameters, we used Euclidean distance, while for the fungal community data, we generated a Bray–Curtis distance matrix. The only significant correlation was between the physical parameters and fungal community composition (Mantel statistic R: 0.1624, *p* = 0.0429), while no significant correlation was found between the chemical and fungal, nor between the chemical and physical parameters. In other words, as samples become more dissimilar in terms of physical parameters, they also become more dissimilar in terms of fungal community composition, while chemical variables appear to change somewhat independently from physical parameters and resident fungi.

## 3. Discussion

The key findings of our paper include that (1) most measured physical and chemical parameters changed predictably during the noble rot process; (2) sampling month during the long harvest period influenced several of these physico-chemical traits, and (3) while keeping to the main trends, cultivars often differed in some physical, chemical, and microbiological aspects [31,33]. In addition, the results largely corresponded to the botrytization phases previously defined based on visual criteria [10,28].

In general, the berry skin parameters indicate that the berry skin becomes weaker, less elastic, and easier to break during the noble rot, likely due to the degradation of cellulose in the plant cell walls by fungi [34]. The only exception of this trend is the slight increase in berry hardness in the last phase in *Furmint*, which may be explained by the excessive drying of the grape berries. It is noteworthy that the berry skin parameters were almost entirely influenced by the botrytization phases and to a small extent by cultivar, but not by sampling month. This means that the visually defined phases can indeed inform growers about the textural characteristics of the berries throughout the harvest season. In addition, the measurable prediction points we detected can help growers to monitor and forecast the onset and development of noble rot, based on the clear drop in textural parameters and the appearance of other grapevine pathogens on the grape berries.

Chemical variables showed a more complex picture than the physical parameters. Sugar content and acidity changed predictably during the noble rot [35,36], the former showing a monotonal increase, while the latter exhibited a peak in phase 3, with a subsequent drop in phase 4, arriving back to the initial levels. Both were affected significantly by the sampling month. Our finding that sugar content was highest late in the harvest season, even among berries representing the same botrytization phase, particularly in phases 3 and 4, suggests that berries considered of the highest quality for making *aszú* wine should be picked in October or November, as even phase 4 berries had much lower (<40 brix) sugar content in September.

Acidity provides an intriguing dilemma. On one hand, there is a clear decrease during the harvest season [37] when berries representing the same botrytization phase are considered. On the other hand, if we consider the noble rot process, phase 1 berries will evolve into phase 3 and phase 4 berries by October and November with acidity remaining fairly constant. Overall, acidity in phase 1 berries in November was much lower than in September and October, making them less suitable as a starting material for high-quality aszú wine. Due to the divergent trends in sugar content and acidity, time of botrytization is important to obtain high quality aszú berries. October seems to be the ideal time for the onset of botrytization of phase 1 berries, which evolve into phase 4 aszú berries by November with very high sugar content and a sufficient acidity level to produce a well-balanced aszú wine.

With respect to the timing of the harvest of fully botrytized phase 4 berries, a compromise needs to be made when sugar content is already high enough and acidity is still adequate in order to make well-balanced or harmonic *aszú* wine. Phase 4 berries in September fall short of the sugar content required for aszú (>50 brix) [5], while their acidity is high. Fortunately for wine makers, the increase in sugar content is steeper than the decrease in acidity in phase 4 berries, making it possible to delay harvest later in the season for top quality *aszú* berries. It is noteworthy that pH appears to be influenced mainly by sampling month and by cultivar and not by noble rot phase. The increase in pH over the harvest season is linked with the decrease in titratable acidity, while the differences between cultivars may partly be attributable to differences in acid composition [38]. It is possible that bacteria, not investigated in this paper, also contribute to the acid content of grape berries, as several genera capable of producing lactic acid and acetic acid have been reported from must and wines in general (e.g., *Acetobacter*, *Gluconobacter*, *Lactobacillus*, *Leuconostoc*, *Oenococcus,* and *Pediococcus*) [39]. Clearly, further studies are needed to explore the metabolomics and the fungal and bacterial microbiome of botrytized grape berries for a more complete characterization of the microorganisms and their functional roles in the noble rot process.

The lack of significant changes in fungal richness among the botrytization phases indicates that the complexity of fungal communities is comparable along the noble rot process. Nonetheless, the significant effect of noble rot phase on the composition of the fungal community suggests some deterministic component of community dynamics that is likely to be related to the predictable physical and chemical changes in the berries [33]. The observed differences in fungal richness and composition among cultivars agree with previous findings on the influence of cultivar on fungal diversity and composition in non-botrytized grapes [25,30]. The increase in fungal richness later in the harvest season could partly be explained by the cooler, more humid conditions in mid- and late-fall that are more favorable to fungal growth in general. Despite the lack of changes in richness during the botrytization process, the significant correlations between physical properties and fungal community composition, as shown by the Mantel test, indicates that berries with more dissimilar physical parameters have more dissimilar sets of fungi. In addition, the observed trend of decreasing beta diversity (community turnover) in phase 4 berries suggests a certain convergence of the fungal community following a partially predictable trajectory as the noble rot progresses. This convergence may be caused by the selection pressure and environmental filtering resulting from the increasing sugar concentration and decreasing water content in the berries [26]. Future research using culture-independent methods are needed to reveal the dynamics of fungal communities during noble rot.

In summary, grape cultivar had weak or no effect on most physical and chemical properties, but it influenced the fungal community and fungi could be responsible for some of the physico-chemical differences observed between the cultivars. Overall, well-balanced sweet wines can be made from these grape cultivars (*Furmint* and *Hárslevelű*) because despite the decrease during the botrytization, acidity level in the last phase of noble rot will be similar to that in the initial phase. It is important to mention that harvest time is primarily important for phase 4 (*aszú* berries) and much less so for partially botrytized grapes and the above-mentioned sensory equilibrium can be best achieved when the *aszú* berries are selected and handpicked berry by berry. Our findings suggest changes in textural properties in the initial stages of botrytization, while differences in chemical parameters are significant in the later stages. Finally, it is still largely unknown as to which microorganisms contribute to the botrytization process and in what roles. Nonetheless, our results indicate that there is more to noble rot than only *Botrytis cinerea* and other microorganisms may play important roles in the *aszú* process.

## 4. Materials and Methods

### 4.1. The Sampling Vineyard

The study was performed in 2017 in the Tokaj wine region in Hungary. The sampling vineyard was located in Mád, named as Betsek (48°11′18.6″ N, 21°19′01.8″ E) where the presence of noble rotted (botrytized) berries is more usual than in other vineyards. The main soil type is yellow clay with soft and white tuff. The vineyard was planted in 1984, the position is south to northwest and the inclination is 5–20%. Planting distance between rows of 2.7 m and within rows of 0.8 m. The pruning type is middle-head cordon with fixed short spurs, and two cultivars were grown, the local white-skinned *Furmint* and *Hárslevelű*. Both cultivars are very suitable for botrytized wine making, which has a very old tradition in this region. In the vineyard, insecticide was used twice (late April and late July) and fungicides were used three times in all years from early-June to late-July (Table S1 in the Supplementary Materials). All sprays were applied with a Lochmann RPS10/70 axial airblast sprayer (Lochmann Plantatech, 10 Nalles, Italy) with a ceramic hollow cone at 0.5 to 1.5 MPa with a volume of 1000 L/hectare. Mechanical weed management was applied by hoeing four or five times annually in the vineyard. Rainfall (mm), daily temperature (mean, minimum, and maximum; °C), humidity (%) and leaf wet (mm) were detected using BASF Defenso agrometeorological station (BASF Hungaria Ltd., Budapest, Hungary) in the vineyard from 1 September until 30 November.

### 4.2. The Sample Collection and Measurement

Four representative botrytization phases were defined before the sampling: (1) healthy berries; (2) starting botrytised, not noble rotten, but purple spotted berries, (3) botrytised, noble rotten and purple berries; and (4) noble rotten raisin berries with latent mycelia [12,28]. Three sampling times were chosen in 2017 (September, October, and November), according to the autumn-period of noble rot development. Based on this, a total of 600 grape berries with symptoms of defined phases were collected randomly in the vineyard in each sampling time and each variety (*Furmint* and *Hárslevelű*). Each berry was detached by cutting its pedicel and visually inspected before analyses. Tests were performed the same day the berries were picked to avoid alterations. The measurements for the appraisal of grape mechanical properties were made on subsamples for each sampling time, phases, variety, and each mechanical test. The remaining berries were crushed and selected for chemical and microbial determinations.

For the testing of physical parameters, a universal testing machine (UTM) TAxT2i Texture Analyzer (Stable Micro System, Surrey, UK) equipped with a HDP/90 platform and a 30 kg load cell was used. Data were evaluated using the Texture Expert Exceed software package. Operative conditions (Table S2 in Supplementary Materials) were chosen based on the research results presented in the literature [13].

For the berry skin hardness test, the berries were placed on the metal plate of the UTM with the pedicel in a horizontal plane so that it was consistently punctured in the lateral face. A 2 mm probe was used, which was enough to puncture the skin without bursting the berry. In the case of the second and third botrytization phase berries, more repetition was needed because the skin of these berries was very thin, according to the noble rot symptoms. For the texture profile analyses (TPA), berries were compressed twice, two seconds apart.

For measuring the basic analytic parameters of sugar content (Brix), titratable acidity (TA), and pH WineScan (FOSS-analytic, Denmark) equipment were used. During the measuring, fresh grape must was analyzed (50 mL), which was cleaned by centrifuge.

For the analyses of microbial diversity, different selective media were used, Chloramphenicol Glucose Agar (CGA), Czapek Dox Agar (CD), and Trypton Glucose Extract (TGE) for fungi growing. The monospore isolates were maintained in Potato Dextrose Agar (PDA) and Yeast Extract Peptone Dextrose Agar (YEPD). For the DNA extraction of fungi, a NucleoSpin Plant II DNA Extraction Kit (Macherey-Nagel, Düren, Germany) was used. The following PCR conditions and primer pairs (Table S3 in Supplementary Materials) were applied for fungi [40,41]. For the purification of PCR products, the QIAquick PCR Purification Kit (Qiagen, Hilden, Germany) was used, and the sequencing was applied by the BaseClear Company, (Leiden, The Netherlands). High-quality sequences were grouped into operational taxonomic units (OTUs) at 97% sequence similarity with USEARCH v. 11 [42]. We assigned OTUs to taxonomic groups based on pairwise similarity searches against the NCBI Nucleotide database. DNA sequences have been deposited at the NCBI GenBank (accession numbers MW349993-MW350005 for filamentous fungi and MW356913-MW356927 for yeasts).

### 4.3. Statistical Analyses

Analysis of variance was applied to all the variables studied. The groups of botrytization phases (1–4), the grape cultivars (*Furmint* and *Hárslevelű*), and the collecting times (September, October, November) were compared according to the Fsk, Esk, Wsk, Berry Hardness (BH), sugar content (Brix), pH, titratable acid (TA) tartaric acid equivalent, and microbial composition. Statistical analyses were performed with the statistical software R (version 4.03) using packages Vegan, mixOmics, and FactoMineR.

Data were filtered for outliers using the Rosner outlier test, scaled and standardized to zero mean and unit standard deviation; normalized data were used where mathematically necessary. To estimate how quantitative dependent variables changed according to the levels of categorical independent variables (e.g., botrytization phase, collecting time, and cultivar) several ANOVA models were tested in one-way, two-way, and two-way-interaction cases. Akaike information criterion (AIC) was calculated to find the information value of each model by balancing the variation explained against the number of parameters used, AICc weights are indicated in Table 1. To find the significant groupwise differences where the 95% confidence interval did not include zero, Tukey’s HSD test was carried out, and the group labelling and spread of the distributions are visualized in Figure 1. It is beneficial to illustrate the multidimensional distribution in reduced space thus principal component analysis (PCA) and partial least square regression (PLSR) were obtained. The scatter of samples in the first two PCs’ plane is plotted in Figure 3 and colored by the noble rotting phases, and the loadings of the quantitative variables can be found in Figure 4.

Differences in fungal community composition among samples were visualized using non-metric multidimensional scaling (NMDS) with *metaMDS* function in the *vegan* R package [43] with Bray–Curtis distance measure. Community turnover (beta diversity) within noble rot phases were calculated with *betadisper* function. The distribution of fungal OTUs among noble rot phases in each cultivar was visualized using the *sna* package [44] based on presence–absence matrices.

The independent quantitative variables were merged into variable groups: texture, analytics, and microbial community. For each group of variables, we performed PerMANOVA to estimate the amount of variation explained by the botrytization phase, sampling month, and cultivar with 9999 permutations. In the texture and analytical case, Euclidean distance matrices were framed; on occasion of microbial community, a Bray–Curtis dissimilarity matrix was performed from the fungal community composition matrix of the samples. To estimate the correlation between the different variable groups, pairwise Mantel tests were carried out using Pearson’s correlation coefficient.

## 5. Conclusions

Until the past decades, the spontaneous appearance of botrytization on grape berries, the *aszú* noble rot process, was considered as the “*gift of nature*”. Even recently, most grapevine-related research has focused on grey rot disease and there have been very few published studies on the noble rot process of grape berries. We present here a comprehensive overview of the changes in the physico-chemical and microbial characteristics of the grape berries during noble rot. Based on the findings presented here, initial stages of botrytization are characterized by marked changes in textural properties that are also visible to the naked eye, while chemical properties changed more markedly in the later stages of the noble rot and are crucial for wine quality. The diversity of fungi detected with our culture-dependent method reveals the microbial complexity of botrytization: there is more to noble rot than only B. *cinerea*, and other microorganisms may also play important roles in the development of *aszú* berries.

## Figures and Tables

**Table 1:** Best fitting ANOVA models for the physical, chemical, and microbiological variables using Akaike information criterion (AIC). Abbreviations are Ph: phases, C: cultivars, Ct: sample collection times, plus sign denotes two-way ANOVA model, asterix indicates interaction model.

	Best Fit Model	AICcwt (%)	*p*-Value
F_sk_	Phases + Cultivars	54	Ph: 2.16 × 10^−9^
			C: 1.47 × 10^−1^
E_sk_	Phases	75	Ph: 9.56 × 10^−6^
W_sk_	Phases + Cultivars	96	Ph: 1.43 × 10^−4^
			C: 6.55 × 10^−3^
BH	Phases	84	Ph: 4.95 × 10^−14^
Sugar	Phases + Collection times	47	Ph: 1.74 × 10^−7^
			Ct: 4.42 × 10^−2^
pH	Cultivars + Collection times	70	C: 1.2 × 10^−4^
			Ct: 2.07 × 10^−2^
TA	Phases + Collection times	100	Ph: 9.26 × 10^−5^
			Ct: 3.44 × 10^−4^
Fungal OTUs	Cultivars * Collection times	95	C: 3.62 × 10^−3^
			Ct: 3.03 × 10^−3^
			C*Ct: 5.02 × 10^−3^

**Table 2:** Distribution of fungal genera among cultivars and noble rot phases, based on similarity searches of DNA sequences generated from cultured isolates against the NCBI Nucleotide database. Abbreviated sample names: F–*Furmint*, H–*Hárslevelű*, with numbers indicating noble rot phases.

Phylum	Order	Genus	F 1	F 2	F 3	F 4	H 1	H 2	H 3	H 4
Ascomycota	Capnodiales	Cladosporium	3	3	0	2	8	4	3	2
Ascomycota	Dothideales	Aureobasidium	4	5	3	3	5	4	2	2
Ascomycota	Pleosporales	Alternaria	2	1	0	0	1	2	1	1
Ascomycota	Pleosporales	Epicoccum	1	0	0	0	1	1	2	0
Ascomycota	Pleosporales	Neoascochyta	0	0	0	0	1	0	0	0
Ascomycota	Chaetothyriales	Exophiala	1	0	0	0	0	0	0	0
Ascomycota	Eurotiales	Aspergillus	0	0	0	0	0	2	2	0
Ascomycota	Eurotiales	Penicillium	0	2	2	2	5	1	2	2
Ascomycota	Helotiales	Botrytis	1	0	1	1	2	4	2	3
Ascomycota	Saccharomycetales	Citeromyces	0	0	0	0	0	0	0	1
Ascomycota	Saccharomycetales	Hanseniaspora	1	5	5	1	1	3	2	0
Ascomycota	Saccharomycetales	Metschnikowia	1	1	1	0	0	1	3	1
Ascomycota	Saccharomycetales	Pichia	0	0	1	0	0	0	0	0
Ascomycota	Saccharomycetales	Torulaspora	0	0	0	1	0	0	0	0
Ascomycota	Saccharomycetales	Zygoascus	0	0	0	0	0	0	0	1
Basidiomycota	Cystobasidiales	Cystobasidium	1	0	0	0	0	0	0	0
Basidiomycota	Sporidiobolales	Rhodotorula	2	2	0	2	4	1	3	1
Basidiomycota	Filobasidiales	Naganishia	0	0	0	0	1	0	0	0
Basidiomycota	Tremellales	Cryptococcus	1	1	1	0	1	1	2	2

**Table 3:** Proportion of variation in physical (texture), chemical (analytics), and fungal community variables explained by noble rot phase, sample collecting time, and grapevine cultivar using permutational multivariate analysis of variance (PerMANOVA).

	Noble Rot Phase		Sampling Month		Cultivar	
Variable	Var. (%)	*p*	Var. (%)	*p*	Var. (%)	*p*
Texture	**57**	**0.001**	**31**	**0.001**	12	0.057
Analytics	**72**	**0.001**	11	0.12	0	0.835
Fungal community	**21**	**0.031**	**22**	**0.014**	**25**	**0.014**

## Supplementary Materials

The following are available online at https://www.mdpi.com/2223-7747/9/12/1809/s1, Figure S1: Scatterplot of the samples in the plane defined by the first two principal components colored by sample collection times, Figure S2: Scatterplot of the samples in the plane defined by the first two principal components colored by cultivars, Figure S3: Scatterplot of the samples in the plane defined by the first two variables of the partial least square statistics, calculated from texture, analytical, and fungal richness variables, colored by botrytization phases, Table S1: Fungicide spray schedules from early April to mid-July in the experimental vineyard (2017, Mád, Betsek Vineyard, Hungary), Table S2: The operative conditions applied for the analyses tests, Table S3: Conditions of DNA extraction.

## References

[1] Kerridge G., Gackle A. (2004). Vines for Wines: A Wine Lover’s Guide to the Top Wine Grape Varieties.

[2] Echikson W. (2004). Noble Rot: A Bordeaux Wine Revolution.

[3] Ribéreau-Gayon P., Dubourdieu D., Donéche B., Lonvaud A. (2006). Other winemaking methods. Handbook of Enology, the Microbiology of Wine and Vinifications.

[4] Magyar I., Soós J. (2016). Botrytized wines–current perspectives. Int. J. Wine Res..

[5] Magyar I., Jackson R.S. (2011). Botrytised Wines. Advances in Food and Nutrition Research Speciality Wines.

[6] Fournier E.P., Gladieux P., Giraud T. (2013). The ‘Dr Jekyll and Mr Hyde fungus’: Noble rot versus gray mold symptoms of Botrytis cinerea on grapes. Evol. Appl..

[7] Holtz G., Coertze S., Williamson B., Elad Y., Williamson B., Tudzymski P., Delen N. (2007). The ecology of *Botrytis* on plant surface. Botrytis: Biology, Pathology and Control.

[8] Elad Y., Vivier M., Fillinger S., Fillinger S., Elad Y. (2016). Botrytis, the Good, the Bad and the Ugly. Botrytis—The Fungus, the Pathogen and Its Management in Agricultural Systems.

[9] Cantu D., Vicente A., Greve L.C., Dewey F.M., Benett A.B., Powell A.L.T. (2008). The intersection between cell wall disassembly, ripening, and fruit susceptibility to *Botrytis cinerea*. Proc. Natl. Acad. Sci. USA.

[10] Hegyi-Kaló J., Holb I., Lengyel S., Juhász Á., Váczy K.Z. (2019). Effect of year, sampling month and grape cultivar on noble rot incidence, mycelial growth rate and morphological type of Botrytis cinerea during noble rot development. Eur. J. Plant Pathol..

[11] Cilibert N., Fermaud M., Joudet J., Rossi V. (2015). Environmental conditions affect *Botrytis cinerea* infection of mature grape berries more than the strain and transposon genotype. Phytopathology.

[12] Carbajal-Ida D., Maury C., Salas E., Siret R., Mehinagic E. (2016). Phisicochemical properties of botrytised Chenin blanc grapes to assess the extent of noble rot. Eur. Food Res. Technol..

[13] Letaief H., Rolle L., Gerbi V. (2008). Mechanical behaviour of winegrapes under compression tests. Am. J. Enol. Vitic..

[14] Rolle L. (2010). Evolution of mechanical variables of winegrapes for icewine production during on-vine drying. Ital. J. Food Sci..

[15] González-Álvarez M., Noguerol-Pato R., González-Barreiro C., Cancho-Grande B., Simal-Gándara J. (2014). Sensory description of sweet wines obtained by the winemaking procedures of raisining, botrytisation and fortification. Food Chem..

[16] Mencarelli F., Tonutti P. (2013). Sweet, Reinforced and Fortified Wines: Grape Biochemistry, Technology and Vinification.

[17] Tofalo R., Chaves-López C., Di Fabio F., Schirone M., Felis G.E., Torriani S., Paparella A., Suzzi G. (2009). Molecular identification and osmotolerant profile of wine yeasts that ferment a high sugar grape must. Int. J. Food Microbiol..

[18] Understanding the Role of Sugar in Wine. https://daily.sevenfifty.com/understanding-the-role-of-sugar-in-wine/.

[19] Cliff M., Yuksel D., Girard B., King M. (2020). Characterization of Canadian ice wines by sensory and compositional analyses. Am. J. Enol. Vitic..

[20] Chen Y., Zhang W., Yi H., Wang B., Xiao J., Zhou X., Xu J., Jiang L., Shi X. (2020). Microbial community composition and its role in volatile compound formation during the spontaneous fermentation of ice wine made from Vidal grapes. Process Biochem..

[21] Bene Z., Magyar I. (2004). Characterization of yeast and mould biota of botrytised grapes in Tokaj wine region in the years 2000 and 2001. Acta Aliment..

[22] Cocolin L., Alessandria V., Dolci P., Gorra R., Rantsiou K. (2013). Culture independent methods to assess the diversity and dynamics of microbiota during food fermentation. Int. J. Food Microbiol..

[23] Blanco-Ulate B., Amrine K.C.H., Collins T.S., Rivero R.M., Vicente A.R., Morales-Cruz A., Doyle C.L., Ye Z., Allen G., Heymann H. (2015). Developmental and metabolic plasticity of white-skinned grape berries in response to *Botrytis cinerea* during noble rot. Plant Physiol..

[24] Azzolini M., Tosi E., Faccio S., Lorenzini M., Torriani S., Zapparoli G. (2013). Selection of *Botrytis cinerea* and *Saccharomyces cerevisiae* strains for the improvement and valorization of Italian passito style wines. FEMS Yeast Res..

[25] Martins G., Miot-Sertier C., Lauga B., Claisse O., Lonvaud-Funel A., Soulas G., Masneuf-Pomarède I. (2013). Grape berry bacterial microbiota: Impact of the ripening process and the farming system. Int. J. Food Microbiol..

[26] Bokulich N.A., Thorngate J.H., Richardson P.M., Mills D.A. (2014). Microbial biogeography of wine grapes is conditioned by cultivar, vintage, and climate. Proc. Natl. Acad. Sci. USA.

[27] Mikusova P., Ritieni A., Juhasová G., Srobárova A. (2010). Contamination by moulds of grape berries in Slovakia. Food Addit. Contam..

[28] Hegyi-Kaló J., Lengyel S., Szalóki N., Szén O., Juhász Á., Váczy K.Z. (2017). Microbial ecology on grape berries of different botrytisation phases. Növényvédelem.

[29] Barata A., Malfeito-Ferreira M., Loureiro V. (2012). The microbial ecology of wine grape berries. Int. J. Food Microbiol..

[30] Bokulich N.A., Ohta M., Richardson P.M., Mills D.A. (2013). Monitoring seasonal changes in winery-resident microbiota. PLoS ONE.

[31] Sato A., Yamane H., Hirakawa N., Otobe K., Yamada M. (1997). Varietal differences in the texture of grape berries measured by penetration tests. Vitis.

[32] Renouf V., Claisse O., Lonvaud-Funel A. (2005). Understanding the microbial ecosystem on the grape berry surface through numeration and identification of yeast and bacteria. Aust. J. Grape Wine Res..

[33] Letaief H., Rolle L., Zeppa G., Gerbi V. (2008). Assessment of grape skin hardness by a puncture test. J. Sci. Food Agric..

[34] Doumouya S., Lahaye C., Maury C., René S. (2014). Physical and physiological heterogeneity within the grape bunch: Impact on mechanical properties during maturation. Am. J. Enol. Vitic..

[35] Pinar A.L., Rauhut D., Ruehl E., Buettner A. (2016). Effects of *Botrytis cinerea* and *Erysiphe necator* fungi on the aroma character of grape must: A comparative approach. Food Chem..

[36] Miklósy É., Kerényi Z. (2004). Comparison of the volatile aroma components in noble rotted grape berries from two different locations of the Tokaj wine district in Hungary. Anal. Chim. Acta.

[37] Deytieux-Belleau C., Geny L., Roudet J., Mayet V., Donéche B., Fermaud M. (2009). Grape berry skin features related to ontogenic resistance to *Botrytis cinerea*. Eur. J. Plant Pathol..

[38] Kiss J., Sass-Kiss A. (2005). Protection of originality of Tokaji Aszú: Amines and organic acids in botrytized wines by high-performance liquid chromatography. J. Agric. Food Chem..

[39] Bartowsky E.J. (2009). Bacterial spoilage of wine and approaches to minimize it. Lett. Appl. Microbiol..

[40] White T.J., Bruns T., Lee S.B., Taylor J.W., Innis M.A., Gelfand D.H., Sninsky J.J., White T.J. (1990). Amplification and direct sequencing of fungal ribosomal RNA for phylogenetics. PCR Protocols: A Guide to Methods and Applications.

[41] Kurtzman C.P., Fell J.W. (1998). The Yeasts.

[42] Edgar R.C. (2010). Search and clustering orders of magnitude faster than BLAST. Bioinformatics.

[43] Oksanen J., Blanchet F.G., Friendly M., Kindt R., Legendre P., McGlinn D., Minchin P.R., O’Hara R.B., Simpson G.L., Solymos P. (2020). Package ‘Vegan’. Community Ecology Package, Version 2.5-7. http://CRAN.R-project.org/package=vegan.

[44] Butts C.T. (2008). Social network analysis with *sna*. J. Stat. Softw..

