# Supplementary material for: Physico-Chemical Characteristics and Culturable Microbial Communities of Grape Berries Change Strongly during Noble Rot Development
Source: Plants (Basel). 2020 Dec 21;9(12):1809. doi: 10.3390/plants9121809 (PMC7766896; doi:10.3390/plants9121809)
Supplement: Supplementary file 1 [file plants-09-01809-s001.pdf]

plants

MDPI

Article (supplementary)

Physico-chemical characteristics and culturable microbial communities of grape berries change strongly during noble rot development

Júlia Hegyi-Kaló[1,2*], Ádám István Hegyi[1] , József Geml[3], Zsolt Zsófi[1], Xénia Pálfi[1], Kálmán Zoltán Váczy[1]

[1]*Food and Wine Research Institute, Eszterházy Károly University, 6 Leányka Street, H-3300 Eger, Hungary*

[2] *Department of Microbiology and Biotechnology, SZIU, 14-16 Somlói street, H-1118 Budapest, Hungary*

[3]*MTA-EKE Lendület Environmental Microbiome Research Group, Eszterházy Károly University, 6 Leányka Street, H-3300 Eger, Hungary,*

* Corresponding author: vaczy.kalman@uni-eszterhazy.hu

Received: date; Accepted: date; Published: date

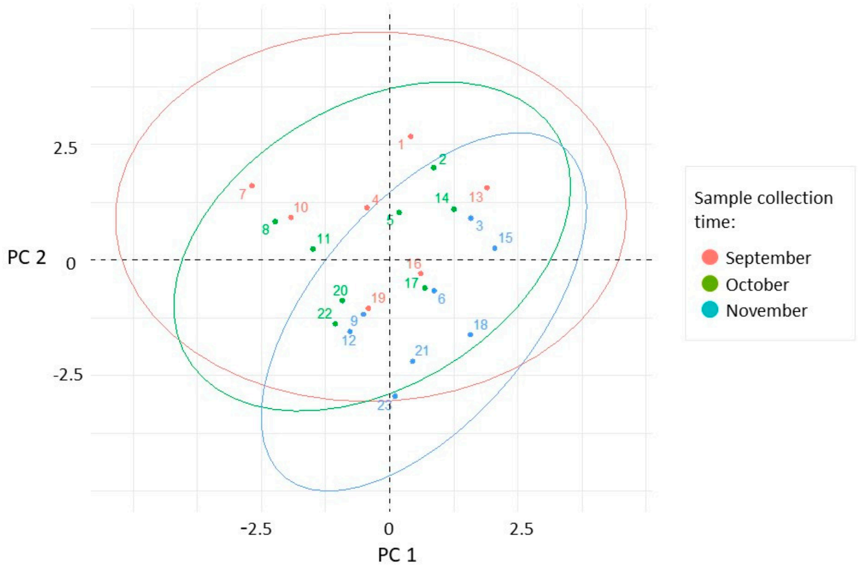

Figure S1: Scatterplot of the samples in the plane defined by the first two principal components colored by sample collection times.

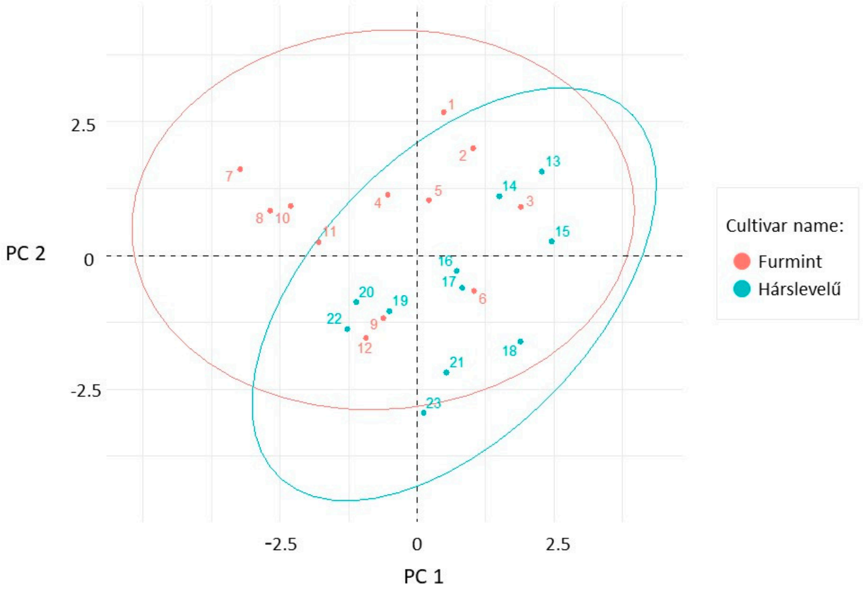

Figure S2: Scatterplot of the samples in the plane defined by the first two principal components colored by cultivars.

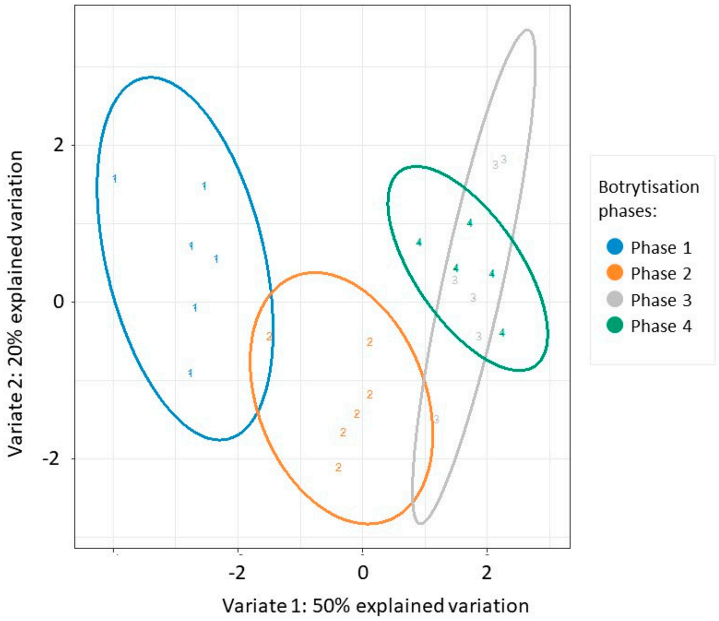

Figure S3: Scatterplot of the samples in the plane defined by the first two variables of the partial least square statistics, calculated from texture, analytical and fungal richness variables, colored by botrytisation phases.

Table S1.: Fungicide spray schedules from early April to mid-July in the experimental vineyard (2017, Mád, Betsek vineyard, Hungary)[a]

Date	Active Ingredients	Trade Name[b]	Dosage
2017			
30 April	abamektin 18 g L^{-1}	Vertimec	0.75 L/ha
10 June	cimoxanil 330 g kg^{-1}, zoxamid 330 g kg^{-1}	Lieto	0.4 kg/ha
	tebukonazol 16%, triadimenol 4%, spiroxamin 25%	Falcon	70 L/ha
	difenokonazol 60 g L^{-1}, cyflufenamid 30 g L^{-1}	Dynali	0.6 L/ha
	elemental sulphur 80%	Kumulus S	0.4 kg/ha
19 June	cimoxanil 330 g kg^{-1}, zoxamid 330 g kg^{-1}	Lieto	0.4 kg/ha
	Tebukonazol 16%,triadimenol 4%, spiroxamin 25%	Falcon	70 L/ha
	difenokonazol 60 g L^{-1}, cyflufenamid 30 g L^{-1}	Dynali	0.6 L/ha
	elemental sulphur 80%	Kumulus S	0.4 kg/ha
	cyflufenamid 4,87 %	Cyflamid	0.35 L/ha
5 July	cimoxanil 330 g kg^{-1}, zoxamid 330 g kg^{-1}	Lieto	0.4 kg/ha
	elemental sulphur 80%	Kumulus S	0.4 kg/ha
26 July	abamektin 18 g L^{-1}	Vertimec	0.75 L/ha

[a] Mechanical weed management (hoing) was applied five times annually in the vineyard.

[b] Vertimec (Syngenta Magyarország Kft, Budapest, Hungary, Lieto (Sumi Agro Hungary Kft, Budapest, Hungary), Falcon (Bayer Hungária Kft, Budapest, Hungary), Dynali (Syngenta Magyarország), Kumulus (BASF Hungary Ltd, Budapest, Hungary), Cyflamid (Sumi Agro Hungary Kft, Budapest, Hungary), Vertimec (Syngenta Magyarország kft, Budapest, Hungary).

Table S2: The operative conditions applied for the analyses tests

Test Type	Probe	Test Speed	Compression	Mechanical Property
Berry skin hardness	2 mm needle, P/2N	1 mm s^{-1}	3 mm	F skin: berry skin break force [N]<br>W_{sk}: berry skin break energy [mJ]<br>E_{sk}: Young's modulus [N/mm]
Texture profile analyses	P/35	1 mm s^{-1}	25% deformation	BH: Berry hardness [N]

Table S3: Conditions of DNA extraction.

Microorganism Type	Primer Pair	Target rRNA Region	PCR Condition
Filamentous fungi	ITS 1F/ITS 4	Fungal ITS1/ITS2	**denature 94 °C 3 min, 94 °C 45 s, annealing 55 °C 1 min, extension 72 °C 2 min, 35 cycles, 72 °C 10 min**
Yeast	NL 1/NL 4	28S D1/D2	**denature 94 °C 3 min, 94 °C 45 s, annealing 55 °C 1 min, extension 72 °C 2 min, 35 cycles, 72 °C 10 min**

© 2020 by the authors. Submitted for possible open access publication under the terms and conditions of the Creative Commons Attribution (CC BY) license (http://creativecommons.org/licenses/by/4.0/).
